# In Sync: Exploring Digital Tools for Sleep and Brain Health in Aging Research

**DOI:** 10.1093/geroni/igaf122.308

**Published:** 2025-12-31

**Authors:** Raeanne Moore, Yeonsu Song, Martin Sliwinski

## Abstract

Understanding the interplay between sleep quality, mood, and cognitive functioning is crucial for older adults with cognitive disorders. This symposium showcases innovative research that harnesses digital health tools to elucidate these complex relationships. We will begin with an overview of new methodologies for measuring sleep and cognition in real-world settings, providing practical examples to help attendees assess the strengths and limitations of these approaches. The discussion will then shift to integrating of digital cognitive assessments and speech analytics to explore cognitive and sleep variability. Developed in collaboration with IBM and McLean Hospital/Harvard, this approach uses AI to monitor fluctuations in cognitive health, capturing the dynamic relationship between sleep and cognitive changes while offering insights into potential signal early signs of cognitive decline. This symposium with include sessions on specific patient populations, starting with dementia care dyads. We will examine daily affective dynamics via ecological momentary assessment, focusing on their impact on subsequent sleep quality for both family care partners and individuals living with dementia. We will also address middle-aged and older individuals living with schizophrenia, who face a heightened risk of dementia. Using wearable sleep trackers and mobile cognitive assessments, we will present evidence of strong correlations between improved sleep metrics—such as later wake time and longer total sleep duration—and enhanced cognitive performance, particularly in attention and memory tasks. This symposium illustrates the transformative potential of digital health tools in advancing research on sleep, mood, and cognition among aging populations, informing targeted interventions and improving brain health outcomes.

